# Supplementary material for: Glypican-3 (GPC-3) Structural Analysis and Cargo in Serum Small Extracellular Vesicles of Hepatocellular Carcinoma Patients
Source: Int J Mol Sci. 2023 Jun 30;24(13):10922. doi: 10.3390/ijms241310922 (PMC10342088; doi:10.3390/ijms241310922)
Supplement: Supplementary file 1 [file ijms-24-10922-s001.zip › ijms-2384958-supplementary.pdf]

Patient#	Age/Sex	Tumor Size (cm)	Tumor Focality	Histologic Type of HCC	Grade	TNM staging	Tumor Extension	Cirrhosis	HCV/HBV
**1**	58/M	2.5	Solitary	Well differentiated	G1	$T_1N_xM_0$	Confined to liver	Cirrhosis/ Steatosis	+/-
**2**	49/M	2	Solitary	Well differentiated	G2	$T_2N_xM_0$	Confined to liver	Cirrhosis	+/-
**3**	68/M	2.1	Solitary	Well differentiated	G2	$T_3N_xM_0$	Involved liver capsule	Cirrhosis	+/-
**4**	69/M	1	Solitary	Well differentiated	G1	$T_1N_xM_0$	Confined to liver	Cirrhosis/Severe Fibrosis	+/-
**5**	60/M	1	Solitary	Well differentiated	G2	$T_1N_xM_0$	Confined to liver	Cirrhosis/Severe Fibrosis	+/-
**6**	53/M	5.5	Solitary	Well differentiated	G3	$T_2N_0M_{N/A}$	Confined to liver	Cirrhosis/Steatosis	+/-
**7**	56/M	1	Solitary	Well differentiated	G2	$T_1N_xM_{N/A}$	Confined to liver	Cirrhosis	+/-
**8**	63/M	3.5	Solitary	Well differentiated	G1	$T_1N_xM_0$	Confined to liver	Cirrhosis	+/-
**9**	65/F	7	Solitary	Well differentiated	G1	$T_1N_xM_{N/A}$	Confined to liver	Cirrhosis	+/-
**10**	55/M	2	Solitary	Well differentiated	G1	$T_1N_0M_{N/A}$	Confined to liver	Cirrhosis	+/-

Supplementary Table S1. Demographic – Pathologic characteristics of HCC patients (N=10)

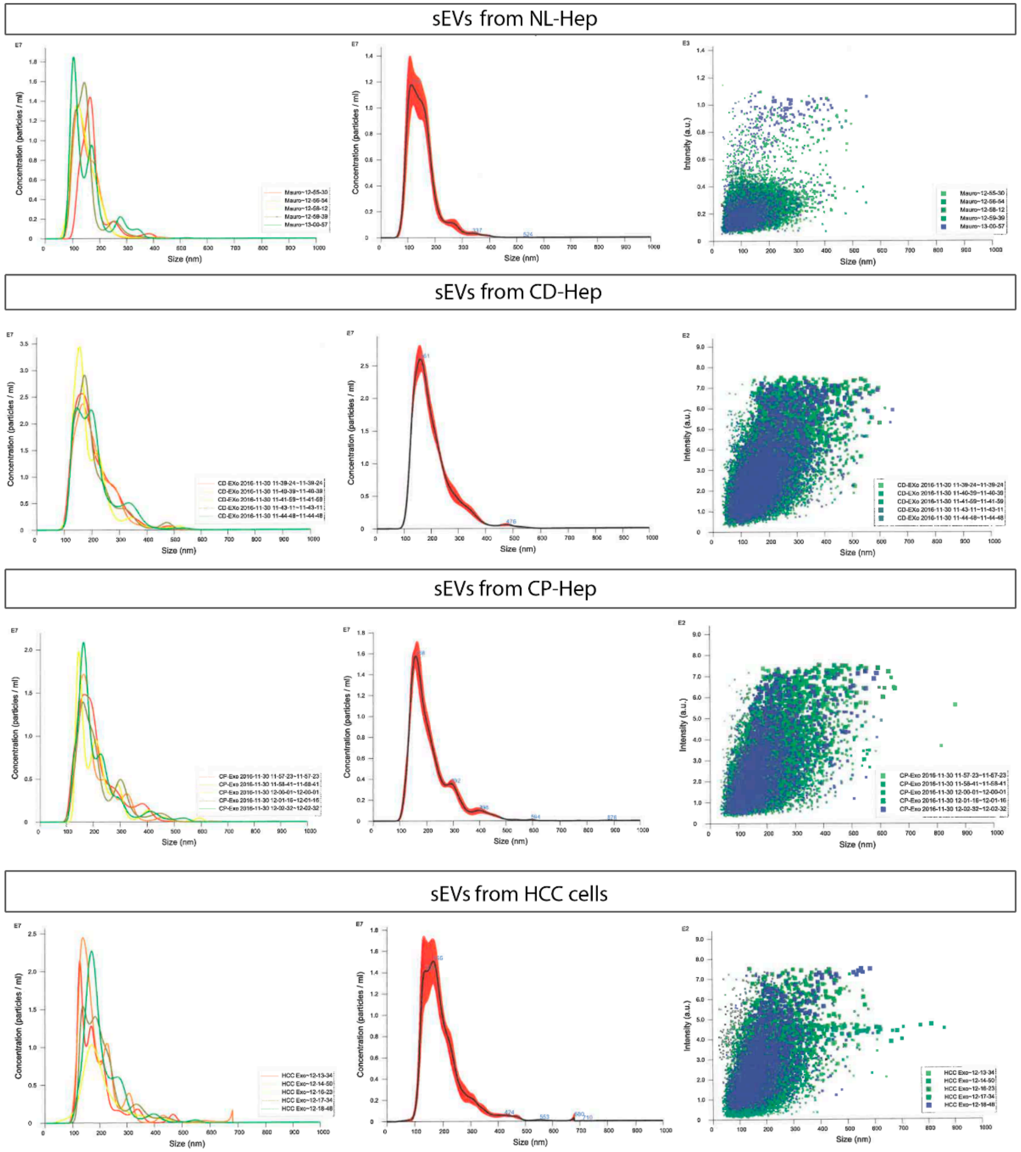

Supplementary Figure S1. Size profiles of small EVs from NTA analysis

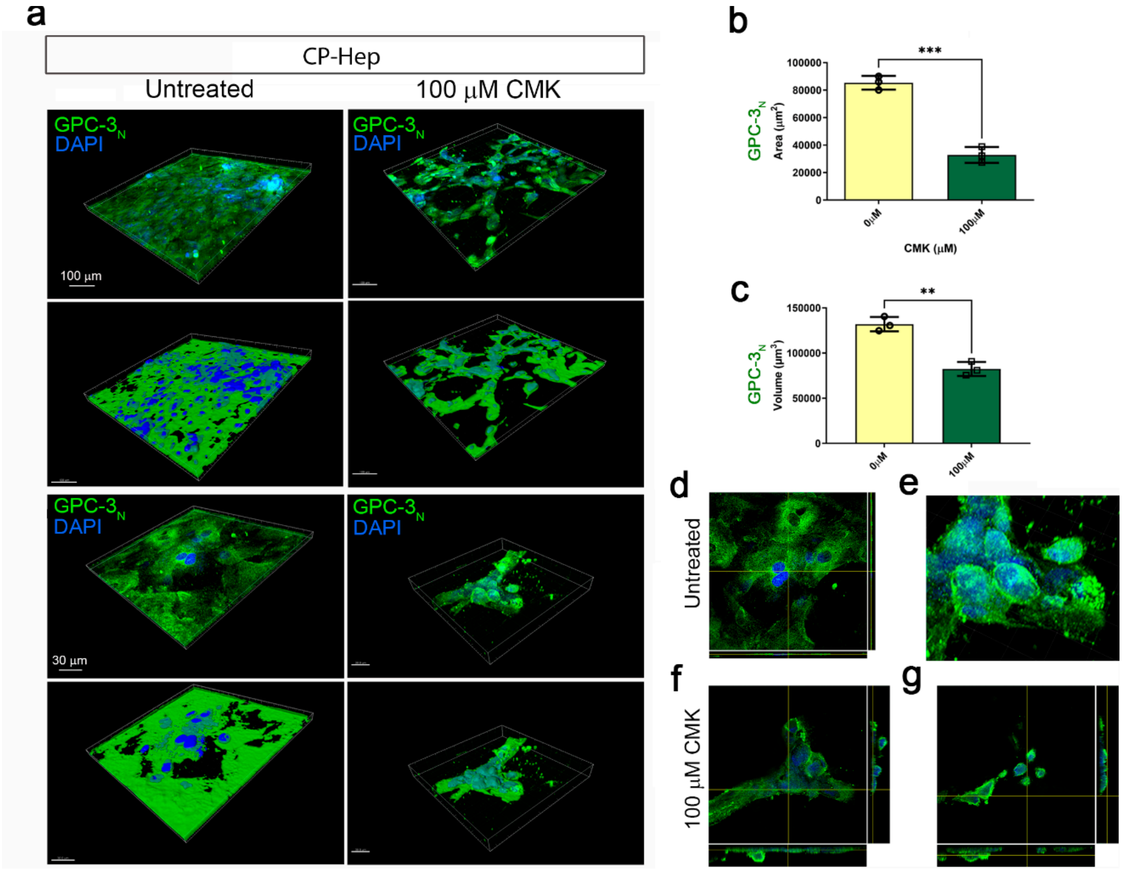

Supplementary Figure S2. (**a**) Representative 3D images of IF GPC-3N (green) in untreated and treated (100µM) CP-Hep. Scale bar 100µm (top panels) and 30µm (bottom panels). (**b**) Quantification of area covered by CP-Hep positive to GPC-3N. Unpaired t-test 0 vs 100µM ***, *p=0.0003*. (**c**) Quantification of cellular volume of CP-Hep positive to GPC-3N. Unpaired t-test 0 vs 100µM ***, *p=0.0015*. (**d**) Representative orthogonal view of CP-Hep untreated and treated with CMK. (**e**) Magnified Z-stack image of hepatocytes exposed to CMK. (**f**) Orthogonal view of median stack frame of cells exposed to CMK. (**g**) Orthogonal view of apical stack frame of cells exposed to CMK.
